# Low-Light Image Brightening via Fusing Additional Virtual Images

**DOI:** 10.3390/s20164614

**Published:** 2020-08-17

**Authors:** Yi Yang, Zhengguo Li, Shiqian Wu

**Affiliations:** 1 Institute of Robotics and Intelligent Systems, School of Information Science and Engineering, Wuhan University of Science and Technology, Wuhan 430081, China; yangyi508@wust.edu.cn; 2 Institute for Infocomm Research, Singapore 138632, Singapore; ezgli@i2r.a-star.edu.sg

**Keywords:** low-light image brightening, intensity mapping function, virtual images, multiscale exposure fusion

## Abstract

Capturing high-quality images via mobile devices in low-light or backlighting conditions is very challenging. In this paper, a new, single image brightening algorithm is proposed to enhance an image captured in low-light conditions. Two virtual images with larger exposure times are generated to increase brightness and enhance fine details of the underexposed regions. In order to reduce the brightness change, the virtual images are generated via intensity mapping functions (IMFs) which are computed using available camera response functions (CRFs). To avoid possible color distortion in the virtual image due to one-to-many mapping, a least square minimization problem is formulated to determine brightening factors for all pixels in the underexposed regions. In addition, an edge-preserving smoothing technique is adopted to avoid noise in the underexposed regions from being amplified in the virtual images. The final brightened image is obtained by fusing the original image and two virtual images via a gradient domain guided image filtering (GGIF) based multiscale exposure fusion (MEF) with properly defined weights for all the images. Experimental results show that the relative brightness and color are preserved better by the proposed algorithm. The details in bright regions are also preserved well in the final image. The proposed algorithm is expected to be useful for computational photography on smart phones.

## 1. Introduction

Exposure time and ISO values are usually carefully tuned by professional photographers to capture well-posed images under various lighting conditions [1,2]. A pairing of short exposure time and a small ISO value can be adopted to reduce motion blur and overexposed regions from an image captured under low-light or backlighting conditions [3]. Since ghost removal is the Achilles’ heel of high dynamic range (HDR) imaging, this method is also efficient to capture images for HDR scenes, especially in the presence of moving objects. However, the visibility of underexposed regions in the captured image is poor. It is necessary to increase the brightness of the underexposed regions in many applications such as tracking ships [4] and capturing CT images [5].

Directly brightening the low-light image is probably the most intuitive and simplest way to improve the visibility of dark regions. Unfortunately, such an intuitive way causes another problem, i.e., relatively bright regions might be saturated and thus lose corresponding details [6]. To address the problem, many single image brightening algorithms were proposed in the past decade. A lightness-order-error measure was proposed in [7] to achieve overenhancement and preserve natural illumination. Based on an observation that an inverted low-light image looks like a haze image, Dong et al. [8] designed an interesting single image brightening algorithm by dehazing the inverted low-light images. The algorithm was further improved in [9] by first segmenting the input image and then adaptively denoising different segments. Even though the above dehazing-like methods can provide visually pleasing results, the basic model lacks physical explanation. A low-lighting image enhancement (LIME) was proposed in [10] to address such a concern by extending the concept of Max-RGB [11] to the pixel level. Particularly, the illumination of each pixel is first estimated individually by finding the maximum value in R, G, and B channels. Then, a structure prior is imposed on initial illumination as the final illumination map. The enhanced image is finally obtained by using the well-constructed illumination map. The framework on the illumination map estimation [10] is similar to the transmission map estimation in [12]. A weighted variational model was proposed for simultaneous reflectance and illumination estimation (SRIE) in [13]. With the estimated reflectance and illumination, the original image can be enhanced by manipulating the illumination. Two virtual images were produced to directly brighten different regions of the input image. All the virtual images and the input image were fused via a multiscale exposure fusion (MEF) algorithm to produce the desired image in [3,14].

**Figure 1:**
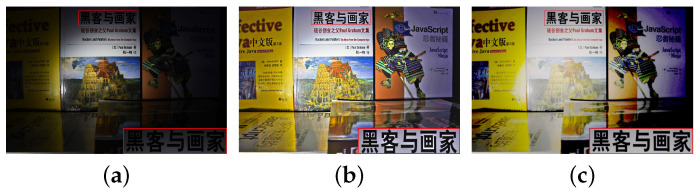
The low-light image and enhanced images are some of technical books about Java. (**a**) is a low-exposed image; (**b**) is obtained by low-lighting image enhancement (LIME) [10]; (**c**) is obtained by the inaccurate intensity mapping function (IMF). Clearly, brightness change is possible issues for the former. Color distortion and noise amplification are possible issues for the latter.

Both the LIME- [10] and the MEF-based algorithms [3,14] assume that the brightness relationships of all the pixels at the corresponding position between the two differently exposed images are linear, i.e., the intensity mapping functions (IMFs) between the differently exposed images are linear functions. This is not always true. As shown in Figure 1b, brightness change is introduced in the red block of the enhanced image if this is not true [15]. To reduce the brightness change, a novel enhancement framework was proposed in [15] by estimating response characteristics of cameras. A reasonable camera response model and its parameters are first determined. Illumination estimation techniques are then adopted to estimate the exposure ratio for each pixel. Finally, the selected camera response model is used to adjust each pixel to the desired exposure according to the estimated exposure ratio map. On the other hand, it is very difficult to estimate the camera response functions (CRFs) using a single input image. Moreover, it is very challenging to use an IMF-based algorithm to brighten a dark image. One possible issue for the IMF-based brightening algorithm is color distortion in the brightened image, as illustrated in Figure 1c. This is because the true relationship of intensity from a dark image to a bright one is a one-to-many mapping for all underexposed pixels [16]. As such, the color distortion could appear in virtual images, especially in underexposed regions of the input image. Furthermore, bright regions could become overexposed when an image is brightened. This is also an issue for single image brightening. This issue is ignored by most deep-learning-based single image brightening algorithms. Another issue is the amplification of noise in the underexposed regions, as shown in Figure 1. The BM3D (Block-Matching and 3D filtering) [17] was used in the LIME as a postprocessing procedure to reduce the noise in the brightened image. As noticed in [15], such a postprocessing method will reduce the efficiency of the image brightening algorithm, especially when it is embedded in mobile devices with limited computational resource. A maximum exposure value is set for pixels with very low illumination in [15] to avoid amplifying noise in the underexposed regions. However, it will lead to the decline of visibility in the very dark regions by setting the maximum exposure ratio. There are also date-driven algorithms that intend to deal with these problems, such as [18,19]. In summary, it is a challenge to keep naturalness while improving the visibility of dark regions in the existing methods. Hence, it is desired to design a simple, single image brightening algorithm which can avoid brightness change, overexposure, and color distortion as well as noise amplification from appearing in the brightened image.

In this paper, a new single image brightening algorithm is introduced to achieve the above objective, and the proposed algorithm is supposed to be embedded in mobile devices such as digital cameras and smartphones. Unlike the algorithm in [15], it is assumed that the CRFs are available. This is not an issue because a set of differently exposed images can be captured in advance to estimate the CRFs by using the algorithm in [20]. To enhance dark regions while preserving the details in bright regions, the enhanced image is obtained from two virtual images and one dark image by using multiscale exposure fusion. Instead of multiplying the dark image by constant ratios, as in [3,14], the virtual images are generated by using the IMFs. Different from [21], the virtual images are generated from a single low-light image, and then the IMFs between the input image and the virtual images are computed from the CRFs. As such, the brightness change is prevented from appearing in the virtual images. The IMFs are used directly to brighten pixels that are not underexposed due to reliability of the IMFs for these pixels. However, color distortions will be brought if the IMFs are directly used to brighten a pixel with one underexposed color channel. To handle this problem, a linear method will be provided to brighten the pixel with at least one underexposed color channel. In other words, the brightened pixel is obtained by multiplying the pixel by a constant ratio. The constant ratio is determined by a least square minimization problem. It is worth noting that the constant ratio is more determined by reliable pixels in order to avoid the effects of underexposed pixels. Besides the color distortion, it is also necessary to avoid amplifying noise in the underexposed regions. A simple edge-preserving denoising method is provided to address the problem. The input image is decomposed into a base layer and a detail layer by using a weighted guided image filter (WGIF), and only the base layer is brightened [16].

All the input image and two virtual images are fused via a state-of-the-art multiscale exposure fusion algorithm, as in [22], to produce the final image. Different pixels usually have different constant ratios. Besides the measurements of contrast, exposure level, and saturation in [23], one more new weighting function is introduced in the proposed algorithm. With the new weighting function, larger weights are given to all the pixels in bright regions of the input image so as to avoid them from being overexposed in the brightened image. Experimental results show that the proposed algorithm outperforms existing single image brightening algorithms. Due to the simplicity of the proposed algorithm, it can indeed be embedded into mobile devices such as an app in smart phones. In summary, three major contributions of this paper are as follows:

1. Propose a simple method to brighten a low-light or backlighting image by fusing additional virtual images;
2. Reduce the possible color distortion of the generated virtual image due to inaccurate IMF;
3. Present a simple method to alleviate the noise in virtual image.

The rest of this paper is organized as follows. Two virtual images are generated in Section 2. All the input image and virtual images are fused together to produce the final image via a multiscale exposure fusion algorithm, described in Section 3. Experimental results are provided in Section 4 to illustrate the efficiency of the proposed algorithm. Finally, the paper is concluded in Section 5.

## 2. Generation of Two Virtual Images

In this section, two virtual images are generated and both of them are brighter than the input image. To avoid the brightness change from appearing in the brightened images, the virtual images are generated by using the IMFs that are computed from the available CRFs as follows:

The IMFs between input image and virtual images are computed via CRFs. The CRFs are generated using the algorithm in [20] by using a large number of differently exposed images.

Let the CRFs $f_{l}\left(\cdot \right)$(l=1,2,3), $Z_{2}$, and $Z_{3}$ be two brightened images from the input $Z_{1}$. Their exposure times are $\Delta t_{1}$, $\Delta t_{2}$, and $\Delta t_{3}$, respectively. Without loss of generality, $\Delta t_{3}$ is equal to $4\Delta t_{2}$ and $\Delta t_{2}$ is equal to $4\Delta t_{1}$. As shown in [21], there is brightness order reversal if the ratios among exposure times are too large. The IMFs between input image and two virtual images can be expressed as follows:

(1) $$\Lambda_{1,i,l}\left(z\right)=f_{l}\left(\frac{f_{l}^{-1}\left(z\right)\Delta t_{i}}{\Delta t_{1}}\right),$$

where $f_{l}^{-1}\left(\cdot \right)$ is the inverse function of the CRF $f_{l}\left(\cdot \right)$.

### 2.1. Generation of Two Intermediate Brightened Images via IMFs

Let the input image be denoted as $Z_{1}$ and $\left[Z_{1,1}\left(p\right),Z_{1,2}\left(p\right),Z_{1,3}\left(p\right)\right]$ be a pixel for three color channels. $Z_{2}$ and $Z_{3}$ are two virtual images which are brightened from the input image $Z_{1}$. Let the IMF from the *c*-th color channel of image $Z_{1}$ to the *c*-th color channel of image $Z_{i}$ be denoted as $\Lambda_{1,i,c}\left(z\right)$. It can be easily verified that the function $\Lambda_{1,i,c}\left(z\right)$ has the following feature:

The function $\Lambda_{1,i,c}\left(z\right)$ is a one-to-many mapping when the value of *z* is smaller than a threshold $\xi_{L}$ and a one-to-one mapping if the value of *z* is above the threshold [16]. Here, the value of $\xi_{L}$ depends on the quality of the camera. The value of $\xi_{L}$ is small for a high-quality camera.

In other words, the IMF from a dark image to a bright one is not reliable if the pixel value is less than the threshold $\xi_{L}$. It is thus necessary to consider the following two cases:

*Case 1*: None of $Z_{1,c}\left(p\right)\left(1\leq c\leq 3\right)$ is less than $\xi_{L}$. The two corresponding brightened pixels are $[\Lambda_{1,2,1}{\left(\left(Z_{1,1}\left(p\right)\right),\Lambda_{1,2,2}\left(Z_{1,2}\left(p\right)\right),\Lambda_{1,2,3}\left(Z_{1,3}\left(p\right)\right)\right]}^{T}$ and $[\Lambda_{1,3,1}{\left(\left(Z_{1,1}\left(p\right)\right),\Lambda_{1,3,2}\left(Z_{1,2}\left(p\right)\right),\Lambda_{1,3,3}\left(Z_{1,3}\left(p\right)\right)\right]}^{T}$, respectively.

*Case 2*: Some of $Z_{1,c}\left(p\right)\left(1\leq c\leq 3\right)$ is (are) less than $\xi_{L}$. The IMFs are not reliable. The color distortion will be brought to the two virtual images if the IMFs are directly used to generate the brightened pixels as in the Case 1. Noise will also be amplified. A piecewise constant method will be first provided to brighten pixels in the underexposed regions of the input image, i.e., when the IMFs are not reliable. An edge-preserving decomposition method will then be provided to avoid noise from being amplified in the brightened images.

### 2.2. Brightening Pixels in Underexposed Regions

A brightening algorithm under the assumption of the linear IMFs generates a brightened pixel by multiplying a pixel to be brightened by a constant. Such a constant brightening algorithm can avoid the color distortion while it causes the brightness change. On the other hand, a brightening algorithm based on the general IMFs can avoid the brightness change while it causes the color distortion for pixels in underexposed regions of the input image. In this subsection, the advantages of both the algorithms are combined to design an algorithm to brighten the pixels in the underexposed region.

Let pixel $Z_{1}\left(p\right)$ be a pixel with a channel value less than $\xi_{L}$. The pixel $Z_{1}\left(p\right)$ will be brightened as $\gamma_{1}Z_{1}\left(p\right)$ and $\gamma_{2}Z_{1}\left(p\right)$, respectively.

The values of $\gamma_{i}\left(i=1,2\right)$ are obtained by solving the following optimization problems:

(2) $$\arg\min_{\gamma_{i}}\left\{\sum_{c=1}^{3}\tilde{w}\left(Z_{1,c}\left(p\right)\right){\left(\Lambda_{1,i+1,c}\left(Z_{1,c}\left(p\right)\right)-\gamma_{i}Z_{1,c}\left(p\right)\right)}^{2}\right\},$$

where the function $\tilde{w}\left(z\right)$ is defined according to different reliability for different pixels. The lower the pixel value, the higher the probability of being affected by noise, which is more likely to bring color distortion in the enhanced image. Therefore, the confidence interval of the pixel with a low value is smaller than the confidence interval of the pixel with a high value. It is defined as

(3) $$\tilde{w}\left(z\right)=\left\{\begin{array}{cc} 1; & \mathrm{if}\;0\leq z<\xi_{L}\\ 128-381h^{2}\left(z\right)+254h^{3}\left(z\right); & \mathrm{if}\;\xi_{L}\leq z<\xi_{U}\\ 128; & \mathrm{otherwise} \end{array}\right.,$$

where $\xi_{U}$ and $\xi_{L}$ are positive constants to determine the reliability of pixel. Their values are chosen by the quality of fused image. The function h(z) is defined as

(4) $$h\left(z\right)=\frac{\xi_{U}-z}{\xi_{U}-\xi_{L}}.$$

Consider the pixel ${\left[Z_{1,1}\left(p\right),Z_{1,2}\left(p\right),Z_{1,3}\left(p\right)\right]}^{T}$ in the input image. It can be shown from Equation (2) that the brightness of the corresponding brightened pixel in the first virtual image is determined by $[\Lambda_{1,2,1}{\left(\left(Z_{1,1}\left(p\right)\right),\Lambda_{1,2,2}\left(Z_{1,2}\left(p\right)\right),\Lambda_{1,2,3}\left(Z_{1,3}\left(p\right)\right)\right]}^{T}$ and in the second virtual image by $[\Lambda_{1,3,1}{\left(\left(Z_{1,1}\left(p\right)\right),\Lambda_{1,3,2}\left(Z_{1,2}\left(p\right)\right),\Lambda_{1,3,3}\left(Z_{1,3}\left(p\right)\right)\right]}^{T}$. In other words, the brightness is determined by the general IMF-based brightening method. As such, the brightness change can be avoided. To further avoid the color distortion, the brightened pixels are generated by using the linear IMF-based method, i.e., the corresponding brightened pixels are ${\left[\gamma_{1}Z_{1,1}\left(p\right),\gamma_{1}Z_{1,2}\left(p\right),\gamma_{1}Z_{1,3}\left(p\right)\right]}^{T}$ and ${\left[\gamma_{2}Z_{1,1}\left(p\right),\gamma_{2}Z_{1,2}\left(p\right),\gamma_{2}Z_{1,3}\left(p\right)\right]}^{T}$, respectively. The resultant brightening algorithm is a piecewise-constant brightening algorithm.

Equation (2) is a classic least squares problem. The values of $\gamma_{i}\left(i=1,2\right)$ can be computed through the method of finding the extremum of partial differential equation. Consequently, the $\gamma_{i}\left(i=1,2\right)$ are computed as

(5) $$\gamma_{i}=\frac{\sum_{l=1}^{3}\tilde{w}\left(Z_{1,c}\left(p\right)\right)Z_{1,c}\left(p\right)\Lambda_{1,i+1,c}\left(Z_{1,c}\left(p\right)\right)}{\sum_{l=1}^{3}\tilde{w}\left(Z_{1,c}\left(p\right)\right)Z_{1,c}^{2}\left(p\right)},$$

and the brightened pixels are given as

(6) $$Z_{i+1}\left(p\right)=\gamma_{i}Z_{1}\left(p\right)\left(i=1,2\right).$$

Clearly, the values of $\gamma_{i}$ are usually different for different pixels. Therefore, the pixels in underexposed regions are brightened using a different method from that in [3,14]. As a result, both the brightness change and the color distortion are being avoided from appearing in the two brightened images.

### 2.3. Noise Reduction of Brightened Images

A dark pixel includes noise. If the pixel $Z_{1}\left(p\right)$ is directly brightened as ${\tilde{\gamma }}_{i}Z_{1}\left(p\right)\left(i=1,2\right)$, the noise is amplified in the brightened pixels. In this subsection, a simple method is provided to address the problem. To reduce noise while preserving details, the pixel $Z_{1}\left(p\right)$ is decomposed as

(7) $$Z_{1}\left(p\right)=Z_{1,b}\left(p\right)+Z_{1,d}\left(p\right)$$

by using an edge-preserving smoothing filter, such as the WGIF in [24]. Here, $Z_{1,b}\left(p\right)$ and $Z_{1,d}\left(p\right)$ are the base layer and the detail layer of pixel $Z_{1}\left(p\right)$, respectively.

The values of ${\tilde{\gamma }}_{i}\left(i=1,2\right)$ are obtained by minimizing the following cost function:

(8) $$\sum_{c=1}^{3}\tilde{w}\left(Z_{1,c}\left(p\right)\right){\left(\Lambda_{1,i+1,c}\left(Z_{1,c}\left(p\right)\right)-Z_{1,c,e}\left(p\right)-{\tilde{\gamma }}_{i}Z_{1,c,b}\left(p\right)\right)}^{2}.$$

Similar to Equation (2), the values of ${\tilde{\gamma }}_{i}\left(i=1,2\right)$ are computed as

(9) $${\tilde{\gamma }}_{i}=\frac{\sum_{c=1}^{3}\tilde{w}\left(Z_{1,c}\left(p\right)\right)Z_{1,c,b}\left(p\right)\left(\Lambda_{1,i+1,c}\left(Z_{1,c}\left(p\right)\right)-Z_{1,c,d}\left(p\right)\right)}{\sum_{c=1}^{3}\tilde{w}\left(Z_{1,c}\left(p\right)\right)Z_{1,c,b}^{2}\left(p\right)},$$

and the brightened pixels are then given as

(10) $$Z_{i+1}\left(p\right)={\tilde{\gamma }}_{i}Z_{1,b}\left(p\right)+Z_{1,d}\left(p\right)\left(i=1,2\right).$$

Since the noise is almost included in the detail layer, the noise is avoided from being amplified by the brightened method (10).

## 3. Fusion of the Input Image and Two Virtual Images

### 3.1. Weights of Three Images

The input image and two virtual images look like three differently exposed images, but they are different from a set of differently exposed images which is captured by a digital camera. Therefore, a different method is adopted to define weights for the three images.

As pointed out in the introduction, a possible issue for the single image brightening is that relatively bright regions might be overexposed in the final image because of the brightening. Clearly, this can be avoided if the details in the relatively bright regions in the final image are from the corresponding regions in the input image. To achieve this objective, two different functions are adopted to define the weights for the three images.

One function is used to determine amplification factors of all the pixels in the first image. To brighten the dark regions while preserving the details in the bright regions, the bright regions are assigned high weights in the low-light image. The weight is defined as

(11) $$w_{1}\left(z\right)=\left\{\begin{array}{cc} 2; & \mathrm{if}\;z>\eta_{U}\\ 1+h_{3}^{2}\left(z\right)\left(3-2h_{3}\left(z\right)\right); & \mathrm{if}\;\eta_{U}\geq z>\eta_{L}\\ 1; & \mathrm{otherwise} \end{array}\right.,$$

where the function $h_{3}\left(z\right)$ is defined as

(12) $$h_{3}\left(z\right)=\frac{z-\eta_{L}}{\eta_{U}-\eta_{L}},$$

and $\eta_{L}$ and $\eta_{U}$ are two constants.

The other is to measure contrast, exposure level, and color saturation of all the pixels in the three images and it is defined the same as in [23]:

(13) $$w_{2}\left(z_{i}\left(p\right)\right)=w_{c}\left(z_{i}\left(p\right)\right)\times w_{s}\left(z_{i}\left(p\right)\right)\times w_{e}\left(z_{i}\left(p\right)\right),$$

where $w_{c}\left(Z_{i}\left(p\right)\right)$, $w_{s}\left(Z_{i}\left(p\right)\right)$ and $w_{e}\left(Z_{i}\left(p\right)\right)$ measure contrast, color saturation, and exposure level of pixel $Z_{i}\left(p\right)$, respectively. Particularly, let $Y_{i}$ be the luminance component of the image $Z_{i}\left(i=1,2,3\right)$. The weight of the pixel $Z_{1}\left(p\right)$ is given as

(14) $$W\left(Z_{1}\left(p\right)\right)=w_{1}\left(Y_{1}\left(p\right)\right)w_{2}\left(Z_{1}\left(p\right)\right),$$

and the weight of the pixel $Z_{i}\left(p\right)\left(i=2,3\right)$ is given as

(15) $$W\left(Z_{i}\left(p\right)\right)=w_{2}\left(Z_{i}\left(p\right)\right).$$

It can be shown in Equations (14) and (15) that the weight of a bright pixel in the input image is much larger than a bright pixel in the two virtual images. As such, the details in the relatively bright regions in the final image are usually from the corresponding regions in the input image. The relatively bright region in the input image is avoided from being overexposed in the final image.

### 3.2. Fusion of Three Images via a GGIF-Based MEF

To get the good fused image, in this subsection, the three images are fused via a gradient domain guided image filtering (GGIF) based on multiexposure images fusion (MEF) [22]. The weight in each level of pyramid is smoothed by the GGIF. To reduce the noise, the guided image is luminance component of input image.

Let $W_{i}^{\left(l\right)}$(i=1,2,3) be weight in *l*-level Gaussian pyramid and GGIF be GG. The weight in *l*-level is computed as

(16) $$W\left({Z_{i}}^{\left(l\right)}\right)=GG\left\{{Y_{i}}^{\left(l\right)},{W_{i}}^{\left(l\right)}\right\},$$

where ${Y_{i}}^{\left(l\right)}$ is the *l*-level Gaussian pyramid of the luminance component of the image $Z_{i}$. The *l*-level fused image is presented as

(17) $${Z_{fused}}^{\left(l\right)}=\frac{\sum_{i=1}^{3}W({Z_{i}}^{\left(l\right)}){Z_{i}}^{\left(l\right)}}{\sum_{i=1}^{3}W({Z_{i}}^{\left(l\right)})}.$$

The final fused image can be reconstructed from ${Z_{i}}^{\left(l\right)}$. Overall, the proposed single image brightening algorithm is summarized as Algorithm 1.
 **Algorithm 1:** Single image brightening and the corresponding CRFs **Input:** a low-light image **Output:** a brightened image **Step 1** Compute IMFs from the available CRFs **Step 2** Generate two virtual images with larger exposure times  *Case1 the value of pixel above 5 do*    Brighten the pixel via Equation (17)  *end*  *Case2 the value of pixel below 5 do*   1. Constructing weight matrix by Equation (2)   2. Decompose input image as base layer and detail layer using WGIF   3. Compute brighten constant $\gamma_{i}$ using Equation (8)   4. Brighten the pixel using Equation (9)  *end* **Step3** Fuse the input image and two virtual images to produce the         final image using Equations (10)–(16).

## 4. Experimental Results

In this section, extensive experimental results are provided to verify the performance of the proposed algorithm. All images are captured by us using a Nikon D7200. It should be noted that the differently exposed images are also captured at the same time.

### 4.1. Generation of Virtual Images

**Figure 2:**
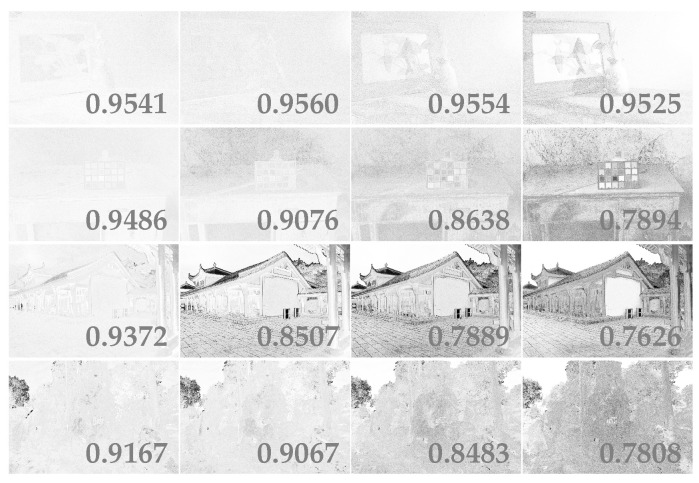
The SSIM (Structural Similarity) index maps of virtual images of different exposure times. The exposure ratios are 2, 4, 8, and 16 from right to left. The SSIMs are in the lower left corner of the images.

**Figure 3:**
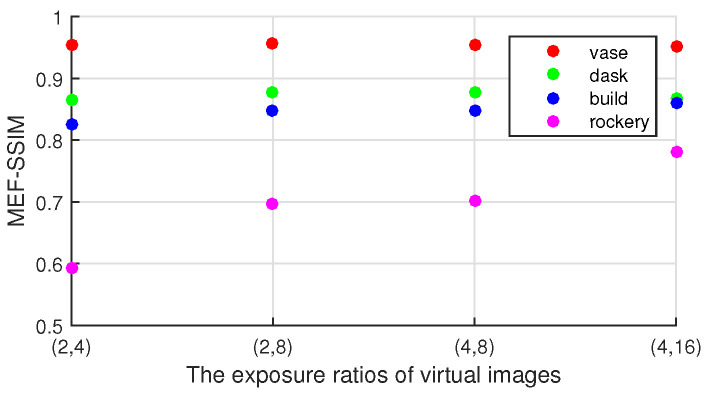
The MEF-SSIMs of enhanced images under different virtual images of different exposure ratios.

In this subsection, the effect of exposure ratios of two virtual images on the quality of enhanced image is analyzed. For the same scene, four groups of enhanced images are generated by fusing virtual images with different exposure ratios. In addition, the quality of different exposure images is also discussed. The ground truths of enhanced image are nine real exposure images and the ground truth of virtual image is the real image with the same exposure time as virtual image. The SSIM (Structural Similarity) index maps are shown in Figure 2. It demonstrates that the higher the exposure ratio of the virtual image to the dark image, the lower the quality of the virtual image is. However, it is found from Figure 3 that the quality of the image quality improved as the virtual image exposure ratios increased. This is because the virtual image with a small exposure ratio is unable to enhance the details in underexposed regions. In the rest of this section, two virtual images are generated with exposure ratios of 4 and 16.

### 4.2. Difference Choices of Parameters

In this subsection, the suitable parameters are selected by a well-used image quality, i.e., the MEF-SSIM in [25]. The test images are three images of different static scenes captured by ourselves. It is worth noting that the exposure ratio of the input image and two virtual images is 1:4:16. Here, the two images with the same exposure times as those of virtual images are also captured for each input image.

**Figure 4:**
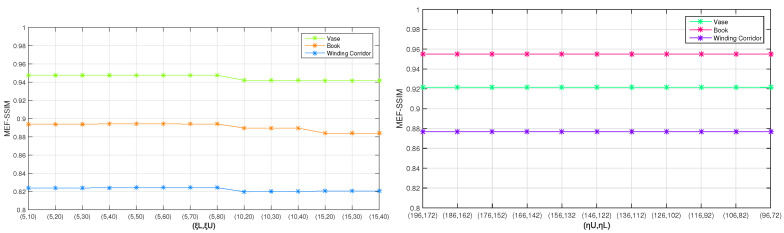
The MEF-SSIM values of the three images under different $\xi_{L}$, $\xi_{U}$, $\eta_{L}$, and $\eta_{U}$ parameters. When $\xi_{L}$ is greater than 5, the SSIM value drops due to the high quality of the camera, as shown on the left. The indexes of different $\eta_{L}$ and $\eta_{U}$ parameters in terms of MEF-SSIM are shown on the right.

When analyzing the influence of $\xi_{U}$ and $\xi_{L}$, the MEF-SSIM is adopted to assess the quality of the virtual images with the largest exposure time. Specially, the reference image is a real image with the same of exposure time as the virtual image. The 14 pairs of $(\xi_{U}$,$\xi_{L})$ and the corresponding MEF-SSIM are shown in Figure 4. Due to the high quality of the camera, the MEF-SSIM of three images decline when $\xi_{L}$ is below 5, as demonstrated in Figure 4. During the determination of $\xi_{L}$, the value of $\xi_{U}$ has little effect on the MEF-SSIM. In this paper, the parameters are set as $\xi_{L}=5,\xi_{U}=60$.

Then, the influence of different pairs of $(\eta_{U}$,$\eta_{L})$ is studied on the fused image. Specially, the reference images are three real, different exposure images with the same exposure times as the three images for fusion. The results of MEF-SSIM are shown in Figure 4. When the values of $\eta_{L}$ and $\eta_{U}$ fluctuate by a small amount, the result will not be affected too much, as shown in Figure 4. In this paper, their values are selected as $\eta_{L}=128,\eta_{U}=160$.

### 4.3. Comparison of the Proposed Algorithm with Existing Ones

In this subsection, we compare our algorithm with seven state-of-the-art image enhancement algorithms, including two data-driven algorithms [18,19] and five model-driven algorithms—LIME [10], NPE [7], LECARM [15], SNIE [13], and Dong [8]. All the test images are taken by the Nikon D7200.

Firstly, the possible color distortion of eight algorithms is analyzed. Since a different light source may introduce luminance change that can affect the overall fluctuation of RGB values in the enhanced image, instead of calculating the absolute difference of RGB value, the angular color between two color vectors respectively produced by enhanced image and ground truth is computed as in [26,27]. As the wavelengths of purple light and blue light are short, the color of image will be biased toward yellow or even red if the exposure time is small. So, the values of pixels on the color checker board are mostly around 128 in the ground truth. Without loss of generality, there is a color checker board in every captured image, and a different color of this board can be regarded as ground truth. The color distortion is expressed as

(18) $$\varepsilon_{n}=\cos^{-1}\left(\frac{Z_{n}\cdot {Z_{n}}^{gt}}{\left|\right|Z_{n}\left|\right|\cdot \left|\right|{Z_{n}}^{gt}\left|\right|}\right),$$

where $Z_{n}$ is the RGB value regarded as the one of the color vectors generated by one enhanced image, and ${Z_{n}}^{gt}$ is the corresponding color vector from the color checker board. It is worth noting that the color vector is averaged around a 3×3 block of the pixel.

**Figure 5:**
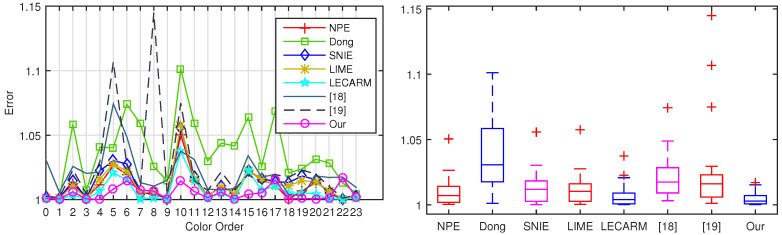
The left box-plot shows the errors of 24 colors from eight algorithms, each error is displayed on the right side in order from left to right, top to bottom.

**Figure 6:**
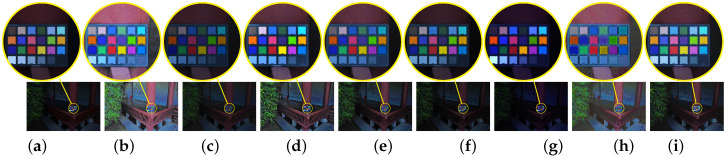
Brighten the color checker board. (**a**) The ground truth of color checker board, (**b**) is by using NPE [7], (**c**) is by using SNIE [13], (**d**) is by using LIME [10], (**e**) is by using Dong [8], (**f**) is by using LECARM [15], (**g**) is by using [18], (**h**) is by using [19], and (**i**) is by using our algorithms.

The 24 color errors of eight algorithms are shown in Figure 5. From the box plots, our median value is the smallest and the maximum value and the minimum value are also the smallest. This means that the 24 errors of our enhanced image follow a narrow distribution and the average is the smallest. It further implies that the proposed algorithm outperforms other seven algorithms in keeping color fidelity. The comparison of the enhanced color checker board is also shown in Figure 6. The figure indicates that the Dong [8] and NPE [7,19] suffer from serious color distortion in multiple color blocks. The LIME [10] makes the image bright enough, but the enhanced image looks too sharp. The LECARM [15] and SNIE [13,18] try reducing distortion at the expense of brightness, however, the first two find it difficult to distinguish the colors of the first three blocks of the last line. By contrast, our algorithm can effectively maintain the authenticity of colors.

Next, the contrast distortion is analyzed in terms of PCQI (Patch-Based Contrast Quality Index) [28], which can predict the overall and local contrast quality of enhanced images. The ground truth is generated by fusing nine different exposure images of the same scene by Kou algorithm [22]. These nine real, different exposure images are taken with a camera from low-exposure to high-exposure, one EV value for each image interval. The assessments of each enhanced algorithm are shown in Table 1. Our algorithm first ranks by contrast quality.

The MEF-SSIM is adopted to further evaluate the performance of eight algorithms. Due to the lack of ground truth, the reference images for the MEF-SSIM are nine real, different exposure images. The nine images contain more information than one dark image, so it is fairer to use this evaluation strategy. The assessment is shown in Table 2. Clearly, our algorithm outperforms all the seven state-of-art algorithms on average.

**Figure 7:**
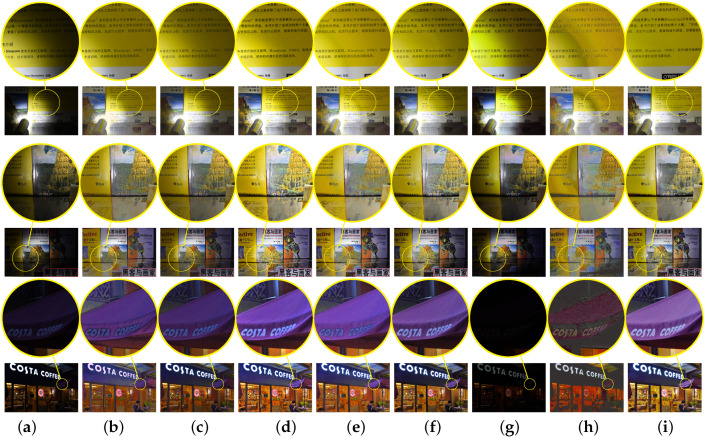
The enhanced results (the images of first and second rows of images are technical books about Java) of eight algorithms. (**a**) shows the input images, (**b**) shows results by using NPE [7], (**c**) shows results by using SNIE [13], (**d**) shows results by using LIME [10], (**e**) shows results by using Dong [8], (**f**) shows results by using LECARM [15], (**g**) is by using [18], (**h**) is by using [19], and (**i**) is by using our algorithms.

**Figure 8:**
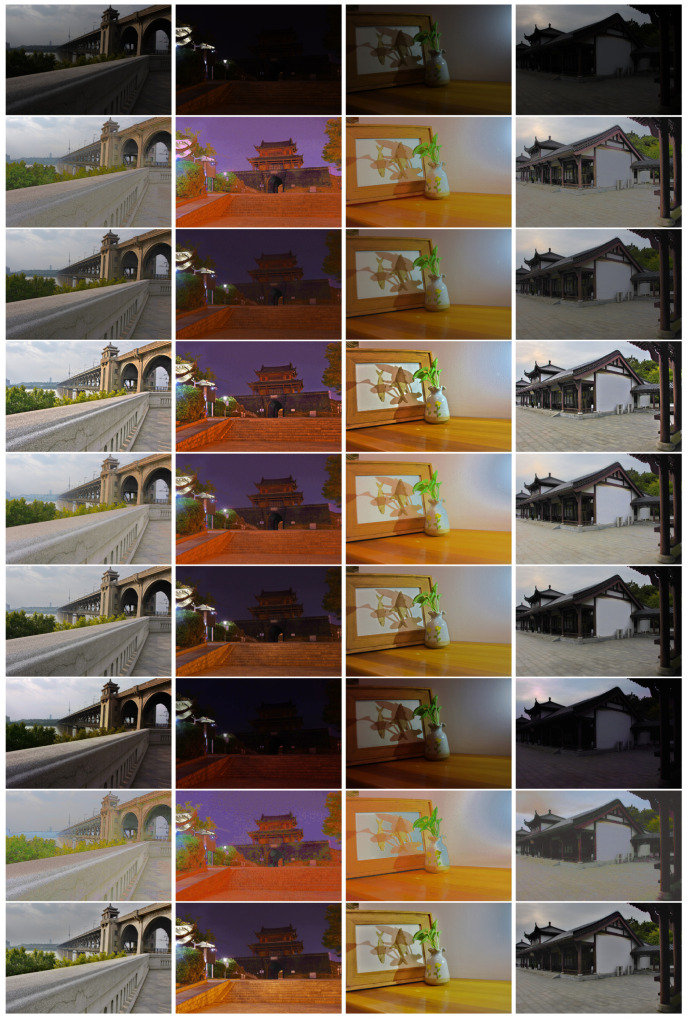
The brightened night images of eight algorithms. The first row shows the input images, the second row is performed using NPE [7], the third row using SNIE [13], the fourth row using LIME [10], the fifth row using Dong [8], the sixth row using LECARM [15], the seventh row using [18], the eighth row using [19], and the last row is by our algorithm.

Besides the objective evaluation, partial enlargements of brighten images are presented in Figure 7 and the large images are also given in Figure 8. Clearly, all eight algorithms can indeed brighten low-light images. However, it can be shown from the second row of Figure 7 that the enhanced image by the LIME [10] looks too sharp. It is demonstrated in the first row of Figure 7 that the LECARM [15] and Dong [8] produce ring artifacts in the enhanced images. Furthermore, there are both color and lightness distortions in the enhanced images by the Dong [8], SNIE [13], and NPE [7,19], as illustrated in the last row of Figure 7. In Figure 8, the enhanced images by the LIME [10] are too sharp and there is color distortion in the first column. There is slight color distortion in the enhanced image by the SINE [13] and the brightness of the enhanced images needs to be improved. All the problems are overcome by the LECARM [15] and the proposed algorithm. Since the proposed algorithm is on top of the CRF, the color is preserved better. This implies that the CRF can be applied to improve the quality of enhanced images.

### 4.4. Efficiency of Noise Reduction via WGIF-Based Smoothing Technique

**Figure 9:**
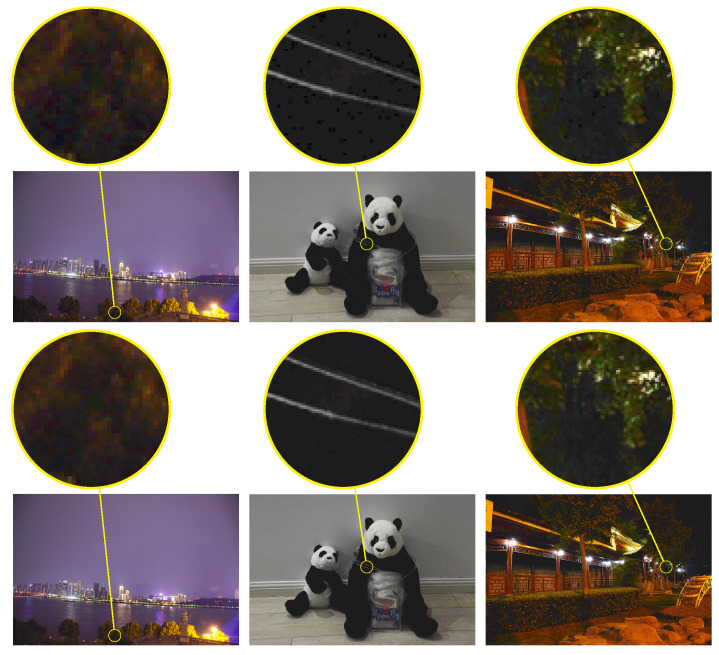
Results on using weighted guided image filter (WGIF)-based noise reduction when the value of a pixel is below 5, and the results of directly amplifying pixels whose value is below 5 by using constant. The first row shows no denoised images, and the second row shown denoised images. Compare the amplified red blocks in the images. The method can effectively reduce noise and preserve details.

In this subsection, efficiency of the noise reduction is evaluated. When the pixel value is less than 5, the constant amplification will lead to noise amplification, as shown in Figure 9.

To reduce the amplified noise and preserve the detail, the base layer of the input image is amplified by Formula (9) while the detail layer is not amplified. As shown in the enlarged yellow block, the noise is indeed reduced in the dark regions, such as the black regions on the panda doll and the shadow of the branches. Moreover, the details of the branches and panda doll have not been weakened. It is worth noting that the BM3D was used in the LIME [10] to reduce the effect of noise. The complexity of the BM3D could be an issue. A maximum exposure ratio was defined in [15] to address the effect of noise. However, setting the maximum exposure ratio will lead to the decline of visibility in the very dark regions. It can even reduce visibility in other regions if it is not defined properly.

It should be pointed out that the WGIF-based smoothing could be omitted if a stack of images is captured. Frame averaging could be used to reduce noise in the dark regions by properly detecting moving regions. If all the corresponding raw images are available, it is not difficult to detect the moving regions.

### 4.5. Comparison of Running Time

**Figure 10:**
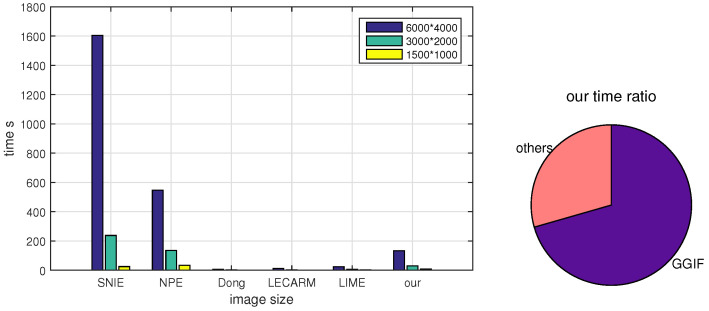
Comparison of the running times between six algorithms. The left shows the times of six algorithms, the right is the percentages on the running time in our algorithm.

The running time of these six algorithms is also compared by testing three image sizes on an AMD 2600X CPU. The results are presented as a bar graph, shown in Figure 10. It should be noted that the two data-driven algorithms are not compared because of the limitations of our hardware. It can be seen in the bar graph that the Dong is the fastest, and our algorithm ranks fourth. Compared with the LECARM and LIME, the running time of our algorithm decreases rapidly with the decrease of image size because the MEF in [22] calculates weights in every image scale. The percentages on the running time of different components in our algorithm is shown in the right. It can be found that the MEF in [22] takes up most of the time. Luckily, the MEF in [22] can be replaced by the simpler MEF in [14] with negligible loss of the MEF-SSIM.

### 4.6. Limitation of the Proposed Algorithm

**Figure 11:**
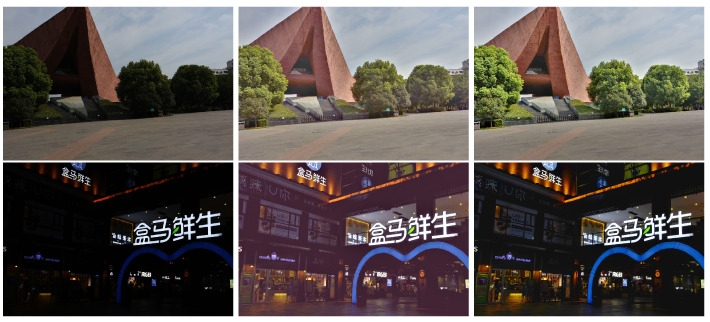
Compare the brightened image (the second row of images is a supermarket in China) by using inaccurate camera response functions (CRFs) and accurate CRFs. The first column shows input images; the second column shows brightened images by using inaccurate CRFs; the last column shows brightened images by using accurate CRFs.

The proposed algorithm outperforms the other five algorithms. However, the proposed algorithm assumes that the accurate CRFs are available. This is not an issue if the proposed algorithm is embedded in a model device. However, this assumption is not true for those images that are downloaded from the Internet. It is very difficult to estimate the accurate CRFs if there is only one image. If the CRFs are not accurate, the IMF between the input image and virtual images are not accurate too. In other words, the brightened image could be a little poor, such as the color distortion in Figure 11. This problem will be studied in our future research.

## 5. Conclusions and Future Remarks

In this paper, we proposed an effective single image brightening algorithm. The proposed algorithm is supposed to be embedded in mobile devices such as digital cameras and smart phones. Two virtually brightened images are generated via intensity mapping functions (IMFs) which are computed by the camera response functions (CRFs). The input image and two virtual images are fused to preserve the natural structure. Since the three images are different from the three differently exposed images that are captured by a camera directly, the weights of these images are specially designed so as to preserve details in bright regions of the input image in the final image. Experimental results show that the proposed algorithm outperforms existing algorithms. The proposed algorithm can be embedded in mobile devices such as digital cameras and smart phones as an app.

It should be pointed out that the CRFs are assumed to be available for the proposed algorithm. Unfortunately, this is not always true. As shown in [29,30], a learning-based method could be adopted to address this issue. The proposed algorithm also assumes that details in bright regions are captured well in the input image. It is also important to enhance details in the bright regions if this assumption is not true. The concept of local inverse tone mapping in [31] could be borrowed to study this problem, and the details could also be preserved using the idea in [32]. The proposed algorithm can be improved by using a data-driven approach. All these problems will be investigated in our future research.

## Figures and Tables

**Table 1:** PCQI of eight different algorithms.

	Book	Building	Coffee	Vase	Winding	Museum	Gate	Pavilion	Market	Bridge	Avg
NPE [7]	0.5756	0.4882	0.4496	0.6245	0.4435	0.4446	0.3568	0.3531	0.5080	0.3262	0.4572
SNIE	0.5312	0.4762	0.4551	0.5966	0.4287	0.4379	0.3650	0.2622	0.4693	0.3150	0.4337
LIME [10]	0.6333	0.4923	0.5584	0.6426	0.5204	0.4380	0.4091	0.3686	0.5195	0.3405	0.4923
LECARM [15]	0.5874	0.4975	0.5173	0.6508	0.4751	0.4461	0.3925	0.2753	0.4945	0.3251	0.4662
Dong [8]	0.5619	0.4595	0.4666	0.5040	0.4329	0.4163	0.3899	0.2990	0.4879	0.3190	0.4337
[18]	0.5210	0.4613	0.4386	0.5802	0.4931	0.4258	0.3897	0.3011	0.4581	0.3589	0.4428
[19]	0.5194	0.4601	0.4067	0.4048	0.4184	0.4019	0.3561	0.2448	0.4610	0.3111	0.3984
Our	0.6229	0.5171	0.5571	0.6756	0.4982	0.4629	0.4350	0.3137	0.5095	0.4260	0.5018

**Table 2:** MEF-SSIM of eight different algorithms.

	Book	Vase	Winding	Museum	Building	Bridge	Coffee	Gate	Pavilion	Market	Avg
NPE [7]	0.8571	0.9237	0.7625	0.6566	0.8149	0.6916	0.7499	0.6285	0.7382	0.7559	0.7579
SNIE [13]	0.8517	0.9405	0.7895	0.6684	0.8402	0.7182	0.7939	0.7097	0.6410	0.7392	0.7692
LIME [10]	0.8730	0.9163	0.8059	0.6391	0.8111	0.6938	0.8398	0.6986	0.7666	0.7797	0.7823
LECARM [15]	0.8897	0.9498	0.8300	0.6729	0.8570	0.7315	0.8459	0.7496	0.6730	0.7711	0.7971
Dong [8]	0.8347	0.8599	0.7313	0.6279	0.8029	0.6431	0.7639	0.6954	0.7143	0.7609	0.7434
[18]	0.7599	0.9135	0.8121	0.6250	0.8109	0.6905	0.7354	0.6821	0.7232	0.7710	0.7524
[19]	0.7257	0.9023	0.7045	0.6134	0.7949	0.6314	0.7144	0.6180	0.7034	0.7216	0.7130
Our	0.9061	0.9525	0.8447	0.6730	0.8601	0.7407	0.8544	0.7513	0.7201	0.7778	0.8081

## References

[1] Zhang L., Deshpande A., Chen X. Denoising vs. deblurring: HDR imaging techniques using moving cameras. Proceedings of the Computer Society Conference on Computer Vision and Pattern Recognition (CVPR).

[2] Hasinoff S.W., Durand F., Freeman W.T. Noise-optimal capture for high dynamic range photography. Proceedings of the Computer Society Conference on Computer Vision and Pattern Recognition (CVPR).

[3] Li Z.G., Zheng J.H. Single image brightening via exposure fusion. Proceedings of the 2016 International Conference on Acoustics, Speech, and Signal Processing.

[4] Chen X., Wang S., Shi C., Wu H., Zhao J., Fu J. (2019). Robust Ship Tracking via Multi-view Learning and Sparse Representation. J. Navig..

[5] Wei W., Zhou B., Połap D., Woźniak M. (2019). A regional adaptive variational PDE model for computed tomography image reconstruction. Pattern Recognit..

[6] Celik T., Tjahjadi T. (2011). Contextual and variational contrast enhancement. IEEE Trans. Image Process..

[7] Wang S., Zheng J., Hu H., Li B. (2013). Naturalness preserved enhancement algorithm for non-uniform illumination images. IEEE Trans. Image Process..

[8] Dong X., Wang G., Pang Y., Li W., Wen J., Meng W., Lu Y. Fast efficient algorithm for enhancement of low lighting video. Proceedings of the IEEE International Conference on Multimedia and Expo.

[9] Li L., Wang R., Wang W., Gao W. A low-light image enhancement method for both denoising and contrast enlarging. Proceedings of the IEEE Conference on Image Processing.

[10] Guo X., Li Y., Ling H. (2017). LIME: Low-light image enhancement via illumination map estimation. IEEE Trans. Image Process..

[11] Land E.H. (1997). The Retinex theory of color vision. Sci. Am..

[12] Li Z.G., Zheng J.H. (2015). Edge-preserving decomposition-based single image haze removal. IEEE Trans. Image Process..

[13] Fu X., Zeng D., Huang Y., Zhang X., Ding X. A weighted variational model for simultaneous reflectance and illumination estimation. Proceedings of the Computer Society Conference on Computer Vision and Pattern Recognition (CVPR).

[14] Li Z.G., Wei Z., Wen C.Y., Zheng J.H. (2017). Detail-enhanced multi-scale exposure fusion. IEEE Trans. Image Process..

[15] Ren Y., Ying Z., Li H., Zheng G.L. (2019). LECARM: Low-light image enhancement using camera response model. IEEE Trans. Circuits Syst. Video Technol..

[16] Li Z.G., Zheng J.H., Zhu Z.J., Wu S.Q. (2014). Selectively detail enhanced fusion of differently exposed images with moving objects. IEEE Trans. Image Process..

[17] Dabov K., Foi A., Katkovnik V., Egiazarian K. (2007). Image denoising by sparse 3D transform-domain collaborative filtering. IEEE Trans. Image Process..

[18] Wang R., Qing Z., Fu C., Shen X., Zheng W., Jia J. Underexposed photo enhancement using deep illumination estimation. Proceedings of the IEEE Conference on Computer Vision and Pattern Recognition.

[19] Wei C., Wang W., Yang W., Liu J. Deep retinex decomposition for low-light enhancement. Proceedings of the British Machine Vision Conference.

[20] Debevec P.E., Malik J. Recovering high dynamic range radiance maps from photographs. Proceedings of the ACM SIGGRAPH.

[21] Yang Y., Cao W., Wu S.Q., Li Z.G. (2018). Multi-scale fusion of two large-exposure-ratio images. IEEE Trans. Image Process..

[22] Kou F., Li Z.G., Wen C.Y., Chen W.H. Multi-scale exposure fusion via gradient domain guided image filtering. Proceedings of the IEEE International Conference on Multimedia and Expo.

[23] Mertens T., Kautz J., Reeth F.V. Exposure fusion. Proceedings of the Conference on Computer Graphics and Applications.

[24] Li Z.G., Zheng J.H., Zhu Z.J., Yao W., Wu S. (2014). Weighted guided image filtering. IEEE Trans. Image Process..

[25] Ma K.D., Zeng K., Wang Z. (2015). Perceptual quality assessment for multi-exposure image fusion. IEEE Trans. Image Process..

[26] Karaimer H.C., Brown M.S. Improving color reproduction accuracy on cameras. Proceedings of the IEEE Conference on Computer Vision and Pattern Recognition.

[27] Schiller F., Valsecchi M., Gegenfurtner R.K. (2018). An evaluation of different measures of color saturation. Vis. Res..

[28] Wang S., Ma K., Yeganeh H., Wang Z., Lin W.S. (2015). A patch-structure representation method for quality assessment of contrast changed images. IEEE Signal Process. Lett..

[29] Cai J.R., Gu S.H., Zhang L. (2018). Learning a Deep Single Image Contrast Enhancer from Multi-Exposure Images. IEEE Trans. Image Process..

[30] Chen C., Chen Q.F., Xu J., Koltun V. Learning to See in the Dark. Proceedings of the IEEE Computer Society Conference on Computer Vision and Pattern Recognition.

[31] Wei Z., Wen C.Y., Li Z.G. (2017). Local inverse tone mapping for scalable high dynamic range image coding. IEEE Trans. Circuits Syst. Video Technol..

[32] Li Z.G., Zheng J.H. (2014). Visual-salience-based tone mapping for high dynamic range images. IEEE Trans. Ind. Electron..

